# Effect of N-acetylcysteine on craving in substance use disorders (SUD): a meta-analysis of randomized controlled trials

**DOI:** 10.3389/fphar.2024.1462612

**Published:** 2024-09-06

**Authors:** Micol Cuocina, Giuseppe Aiello, Pierfelice Cutrufelli, Martina Rampello, Laura Rapisarda, Alessandro Rodolico, Giuseppina Cantarella, Maria Salvina Signorelli, Renato Bernardini

**Affiliations:** ^1^ Department Biomedical and Biotechnological Sciences (BIOMETEC), Section of Pharmacology, University of Catania, Catania, Italy; ^2^ Clinical Toxicology Unit, University Hospital of Catania, Catania, Italy; ^3^ Psychiatry Unit, Department of Clinical and Experimental Medicine, University of Catania, Catania, Italy; ^4^ Technical University of Munich, TUM School of Medicine and Health, Department of Psychiatry and Psychotherapy, Munich, Germany

**Keywords:** craving, N-acetylcysteine, SUD, addiction, drugs

## Abstract

**Background:**

N-acetyl cysteine (NAC) appears promising as a treatment in patients with substance use disorder (SUD) as it helps rebalance glutamate levels in the central nervous system (CNS). Basal concentrations of glutamate are indeed reduced in SUD patients but increased during craving.

**Materials and Methods:**

We conducted a systematic review and meta-analysis of randomized controlled trials (RCTs). We assessed whether NAC reduce craving rating as compared to a placebo in SUD patients. Secondary outcomes were withdrawal symptoms (WS), side effects (SE) and drop-outs. Estimates are presented as standardized mean differences (SMD) or risk ratio (RR) with 95% confidence interval (CI).

**Results:**

Eleven RCTs were included. NAC reduced craving rating (SMD -0.61 (−1.17, −0.06), *p* = 0.03, I2 = 85%), with no differences in the subgroup analysis according to the drug addiction (alcohol, cocaine, poly-drugs, amphetamine, nicotine) (*p* = 0.98). Among the secondary outcomes, for WS data showed no significant difference between groups (SMD -0.18 (−0.43, 0.08), *p* = 0.17); for SE no substantial difference was observed between the two treatment groups (RR = 1.06 (0.89–1.27), *p* = 0.52, I2 = 0%); for dropouts the results are in favor of the placebo but no statistically significant (RR 1.17 (0.85, 1.61), *p* = 0.34; I2 = 0%).

**Conclusion:**

NAC seem to reduce craving rating in SUD patients, but evidence is weak. More studies are needed to confirm this finding.

## 1 Introduction

According to the United Nations Office on Drugs and Crime (UNODC), approximately 39.5 million people globally were estimated to be suffering from Substance Use Disorder (SUD) in 2021 (UNODC, 2023). SUD is a neuropsychiatric condition characterized by compulsive and uncontrollable substance-seeking behavior, resulting in significant long-term consequences due to changes in brain chemistry and morphology (Kalivas, 2004). It represents a major public health issue, often accompanied by multiple medical complications that negatively impact an individual’s quality of life, professional performance, personal relationships, and overall wellbeing (Fuster et al., 2024).

As a chronic medical condition, SUD requires early diagnosis and intervention to mitigate drug-related issues. The fifth edition of the Diagnostic and Statistical Manual of Mental Disorders (DSM-5-TR) defines SUD as a cluster of cognitive, behavioral, and physiological symptoms indicating continued substance use despite significant substance-related problems (Association, 2022). Craving, characterized by an intense desire or urge for the substance, is a key feature of SUD and is often predictive of subsequent substance use and potential treatment discontinuation (Cless et al., 2023).

One of the major challenges in treating SUD is managing the intense craving experienced by individuals. This craving is linked to pathological alterations in brain plasticity mechanisms, particularly in areas associated with craving and relapse (Kalivas, 2004). Dysfunctional cortical-hippocampal-striatal prefrontal circuits interconnected by glutamatergic signaling have been implicated in SUD (Smaga et al., 2019). Preclinical research suggests that repeated exposure to addictive substances leads to changes in extracellular glutamate concentrations, including reduced expression of glial glutamate transporter 1 (GLT-1) and the cystine-glutamate exchange system/antiporter in regions such as the nucleus accumbens and prefrontal cortex (Wydra et al., 2013; Smaga et al., 2020).

Clinical studies have demonstrated that individuals with SUD exhibit altered glutamate homeostasis, characterized by reduced basal glutamate concentrations and increased levels during induced craving. Modulating cystine-glutamate exchange has emerged as a potential strategy to decrease excitatory glutamatergic transmission following drug use. N-acetylcysteine (NAC) has been identified as a promising target for new SUD pharmacotherapies due to its role in rebalancing glutamate levels (Engeli et al., 2021; Smaga et al., 2021).

NAC, commonly used for its mucolytic effects and as an antidote for acetaminophen overdose (dos Santos Tenório et al., 2021), acts as a precursor to glutathione (GSH), replenishing intracellular GSH pools depleted under conditions of oxidative stress, drug detoxification, or other scenarios leading to GSH deficit (Frye et al., 2019; Samuni et al., 2013). It modulates glutamatergic pathways through the system xc−, enhancing cystine–glutamate exchange and reducing extracellular glutamate levels. Preclinical studies have shown that NAC can prevent drug intake escalation, behavioral sensitization, and cocaine-induced reinstatement (Dean et al., 2011; Olive et al., 2012; Madayag et al., 2007).

Recent clinical studies have confirmed these preclinical findings, demonstrating that NAC can reduce elevated glutamate levels in the dorsal anterior cingulate cortex (dACC) and improve impulsivity in cocaine-dependent patients. Furthermore, NAC has been shown to decrease cocaine-seeking behavior during cocaine-primed sessions without affecting behavior during placebo-primed sessions (Woodcock et al., 2021; Schmaal et al., 2012). These findings highlight NAC’s potential as a treatment for addiction, addressing underlying glutamatergic abnormalities and modulating brain regions involved in addictive behaviors.

This meta-analysis aims to evaluate the efficacy of N-acetylcysteine in reducing drug craving compared to placebo across a range of substances, including nicotine, cannabis, cocaine, amphetamines, and alcohol, by consolidating data from numerous randomized controlled trials (RCTs).

## 2 Methods

### 2.1 Search strategy, selection criteria and data extraction

We conducted a systematic search and meta-analysis of RCTs comparing N-acetyl cysteine (at any dosage) with placebo in individuals with SUD (nicotine, cannabis, cocaine, heroin, amphetamine, morphine, opioid, and alcohol).

**FIGURE 1:**
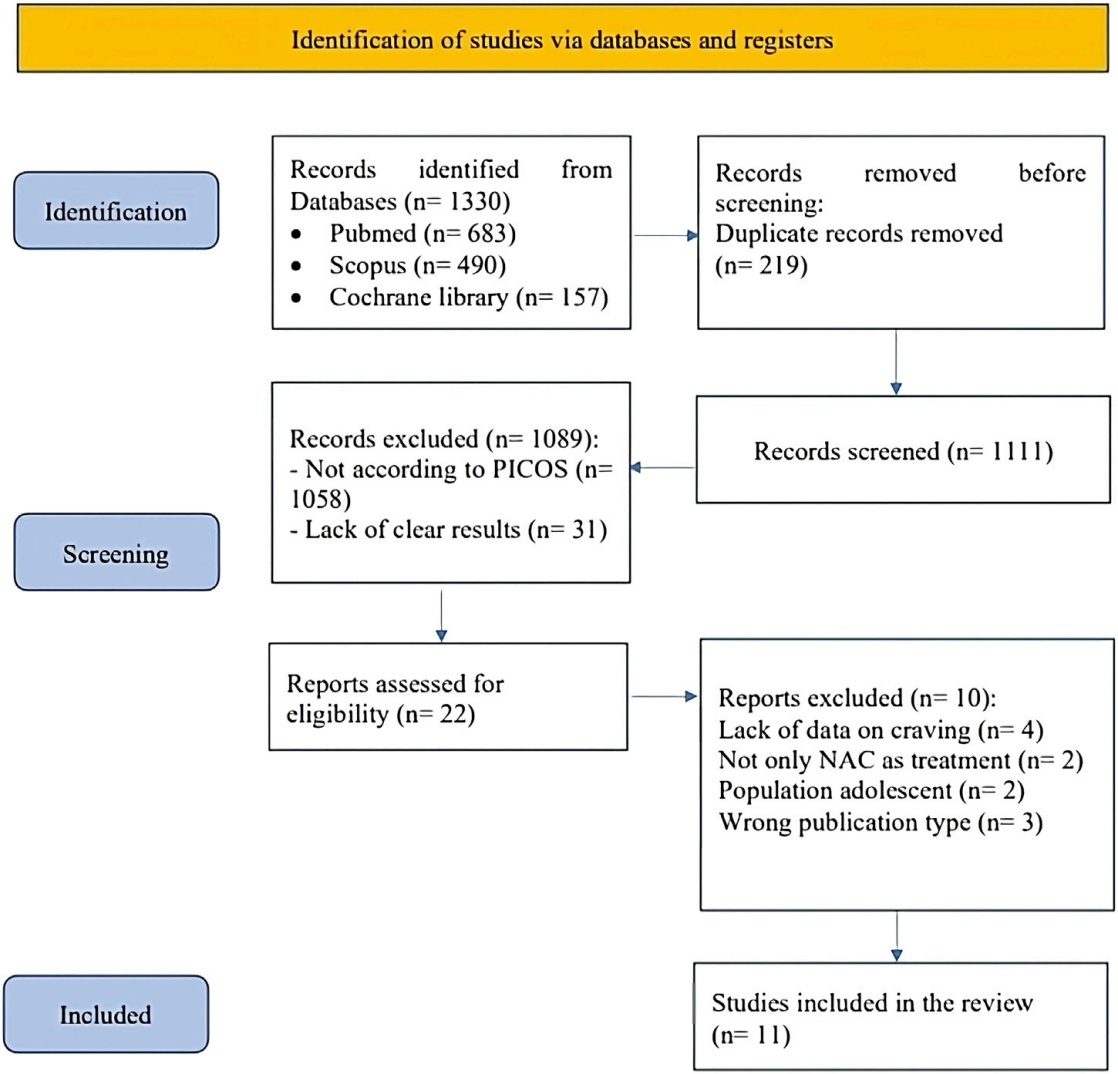
PRISMA flowchart graphically describes the process of screening, selection and inclusion of articles. Abbreviation. PRISMA: Preferred Reporting Items for Systematic reviews and Meta-Analyses.

The protocol was registered on PROSPERO database (CRD 42023435574). The detailed process of study selection is illustrated in the PRISMA Flowchart (Figure 1). The metanalysis was conducted according to the PRISMA guidelines (Page et al., 2021) and a PRISMA checklist is provided as Supplementary Material. Studies were included according to the PICOS approach (Supplementary Material). For the primary analysis, we considered only RCTs including individuals with SUD treated with N-acetyl cysteine at any dosage compared to Placebo. Primary outcome was Craving rating, while secondary outcomes were withdrawal symptoms, adverse events (number of non-serious adverse events, number of serious adverse events) and dropouts. Studies on the pediatric/adolescent population and those where NAC was administered in addition to another drug were excluded. We included only manuscripts published in English. Relevant articles were identified with a computerized search of the MEDLINE (PubMed), Scopus and Cochrane Library databases from inception until February 29^Th^, 2024, using the following Medical Subject Headings terms (“Acetylcysteine” [MeSH] AND (“Substance-Related Disorders” [MeSH] OR “Marijuana Abuse” [MeSH] OR “Tobacco Use Disorder” [MeSH] OR “Morphine Dependence” [MeSH] OR “Heroin Dependence” [MeSH] OR “Opioid-Related Disorders” [MeSH] OR “Cocaine-Related Disorders” [MeSH] OR “Amphetamine-Related Disorders” [MeSH] OR “Alcoholism” [MeSH]). Study selection for determining eligibility for inclusion in the systematic review and data extraction was performed independently by four reviewers (GA, MC, PC, MR) with the supervision of another author (AR). Discordances were resolved through consensus at a meeting of the four authors and by involving senior authors (RB, GC, AR). A manual search was conducted independently by two authors (MC, GA) to explore the reference lists for the findings of the systematic search. Two additional authors (LR, MR) independently double-checked the data collected.

When it became necessary, the authors of the study were contacted directly for clarifications. In the absence of tabular or in-text data, information was extracted from graphs using WebPlotDigitizer (https://automeris.io/WebPlotDigitizer).

### 2.2 Statistical analysis

Meta-analysis was conducted using the Cochrane Review Manager version 5.4 (The Cochrane Collaboration). Dichotomous outcomes were analyzed as risk ratio (RR) with 95% confidence interval (CI) using the Mantel-Haenszel method. Continuous outcomes were analyzed as mean difference (SMD) with 95% CI, *p* values were considered significant if < 0.05. Heterogeneity across studies was estimated by I^2^ and Tau^2^. Due to high statistical heterogeneity, the random-effect model was used. Potential publication bias was assessed by inspection of the funnel plot (Supplementary Material).

### 2.3 Quality assessment

The methodological quality of the included RCTs, specifically for the craving rating, was evaluated using the Cochrane RoB2 tool., which incorporates the following domains: randomization process, deviation from intended interventions, missing outcome data, measurement of the outcome and selection of the reported result. Grading of the evidence was performed according to the recommendations of the Grading of Recommendations Assessment, Development and Evaluation working group by two authors (GA and MC) using the GRADEpro software available at https://gdt.gradepro.org/(accessed on April 16^th^ 2024).

### 2.4 Outcomes

We primarily compared the reported efficacy of N-acetylcysteine in craving rating in addicted patients. Secondary outcomes evaluated withdrawal symptoms, side effects and drop out.

### 2.5 Subgroup analyses and Trial Sequential Analysis (TSA)

We conducted a subgroup analysis based on the type of addiction within the study population for each Randomized Controlled Trial (RCT) (nicotine, cannabis, cocaine, amphetamines, alcohol and poly-drugs). We conducted a Trial Sequential Analysis (TSA) on the primary outcome utilizing the TSA Software (Copenhagen Trial Unit’s TSA Software^®^; Copenhagen, Denmark). The determination of the ‘information size’ (sample size required to ensure reliable conclusions) was made, assuming a type 1 error of 5% and a power of 90%. Additional information regarding TSA methodology and its interpretation can be found elsewhere (Sanfilippo et al., 2021; Kang, 2021).

## 3 Results

Our systematic search identified 683 studies via PubMed, 490 via Scopus and 157 from Cochrane library; after removing 219 duplicates, a total of 1,111 abstracts were screened. Of these, 1,058 were excluded due to not being focused on the topic of interest and 31 due to lack of clear results. After title and abstract selection, only 22 studies were judged to be of potential interest for our quantitative analyses. However, when considering the study design as per the PICOS criteria, we included only 11 RCTs in the review (LaRowe et al., 2013; Knackstedt et al., 2009; Morley et al., 2023; McKetin et al., 2021; Schulte et al., 2018; Back et al., 2016; Froeliger et al., 2015; Mousavi et al., 2015; Yoon, 2013; Schmaal et al., 2011; LaRowe et al., 2006) (As shown in Figure 1). One of the studies with a large sample size, Gray et al. (2017) (Gray et al., 2017), could not be included in the final analysis due to the absence of craving data post-treatment.

The characteristics of the included studies are shown in Table 1. Of the eleven RCTs included, the most recent one (Morley et al., 2023) enrolled 42 patients and curiously the results are the only ones not to be in line with all the other studies conducted so far. The study with the largest population (n = 153) (McKetin et al., 2021) is also relatively recent and shows results that are also not in line with the other studies. The overall results of our meta-analysis are shown in Table 2.

**TABLE 1:** Characteristics of the populations and the interventions in the included studies selected for meta-analysis.

Studies	Outcomes reported	Substance	Intervention	Sample	Sex		Age (mean)	Craving Scale used	Dosage	Duration of the intervention (weeks)
					M	F				
Morley, 2023 Alcohol and Alcoholism	Craving, dropouts	Alcohol	NAC	21	13	8	48.60 (11.70)	PACS	2,400 mg/die	4
			Placebo	21	13	8	48.50 (9.90)			
McKetin, 2021 EClinicalMedicine	Craving, WS, dropouts	Methamphetamine	NAC	76	45	31	37.5 (8.4)	CEQ	2,400 mg/die	12
			Placebo	77	46	31	37.9 (7.9)			
Schulte, 2018 Addictive Behaviors	Craving	Poly-drug	NAC	17	17	0	41.35 (9.77)	VAS	2,400 mg/die	4
			Placebo	21	21	0	32.19 (7.87)			
Back, 2016 J Clin Psychiatry	Craving, AE, dropouts	Poly-drug	NAC	13	12	1	48.20 (8.60)	VAS	2,400 mg/die	8
			Placebo	14	14	0	49.90 (8.10)			
Froeliger, 2015 Drug and Alcohol Dependence	Craving, WS	Nicotine	NAC	8	6	2	35.00 (14.40)	8-point Likert Scale Questionnaire	2,400 mg/die	0.6
			Placebo	8	5	3	38.00 (9.60)			
Mousavi, 2015 Archives of Iranian Medicine	Craving, AE, dropouts	Amphetamine	NAC	11	9	2	29.90 (4.70)	CCQ-Brief	1,200 mg/die	8
			Placebo	12	10	2	28.50 (5.10)			
LaRowe, 2013 The American Journal on Addictions	Craving, AE, dropouts	Cocaine	NAC	73	55	18	43.40 (9.51)	BSCS	1,200 or 2,400 mg/die	8
			Placebo	38	28	10	42.80 (8.70)			
Yoon 2013	Craving, AE, dropouts	Alcohol	NAC	22	20	2	50.1 (11.3)	PACS	3,600 mg/die^a^	8
			Placebo	24	22	2	56.5 (7.0)			
Schmaal, 2011 Eur Addict Res	Craving, WS AE	Nicotine	NAC	10	4	6	21.40 (2.07)	QSU-B	3,600 mg/die	1
			Placebo	12	5	7	20.25 (1.14)			
Knackstedt, 2009 BIOL PSYCHIATRY	Craving, WS, dropouts	Nicotine	NAC	16	9	7	51.30 (10.10)	QSU-B	2,400 mg/die	4
			Placebo	17	10	7	48.60 (10.50)			
LaRowe, 2006 The American Journal on Addictions	Craving, WS	Cocaine	NAC	13	6	7	37.10 (7.60)	5 items Likert ten-point	600 mg/die	0.4
			Placebo	13	6	7	37.10 (7.60)			

Abbreviations: *PACS*, Penn Alcohol Craving Scale; *WS*, Withdrawal Symptoms; *CEQ*, Craving Experience Questionnaire; *VAS*, Visual Analogue Scale; *AE*, Adverse Effects; CCQ-Brief, Cocaine Craving Questionnaire-Brief; *BSCS*, Brief Substance Craving Scale; *QSU-B*, Questionnaire for Smoking Urges-Brief.

^a^ N-acetylcysteine 900 mg/day for week 1, 1800 mg/day for week 2, 2,700 mg/day for week 3 and 3,600 mg/day for week 4.

**TABLE 2:** Summary of the results of the primary and secondary outcomes comparing treatment with NAC vs. Placebo (control group). Abbreviations: SMD, Standardized Mean Difference; RR, Risk Ratio; CI, Confidence Interval.

	Studies	Patients (n)	SMD or RR (95% CI)	p-value	Heterogeneity	
					I2	p-value
Craving rating	11	446	SMD -0.61 (−1.17, −0.06)	0.03	85%	<0.00001
Withdrawal symptoms	5	245	SMD -0.18 (-0.43, 0.08)	0.17	0%	0.56
Adverse events	5	250	RR 1.06 (0.89, 1.27)	0.52	0%	0.52
Drop-out	8	490	RR 1.17 (0.85, 1.61)	0.34	0%	0.91

Abbreviations: *SMD*, standardized mean difference; *RR*, risk ratio; *CI*, confidence interval.

### 3.1 Primary outcome

**FIGURE 2:**
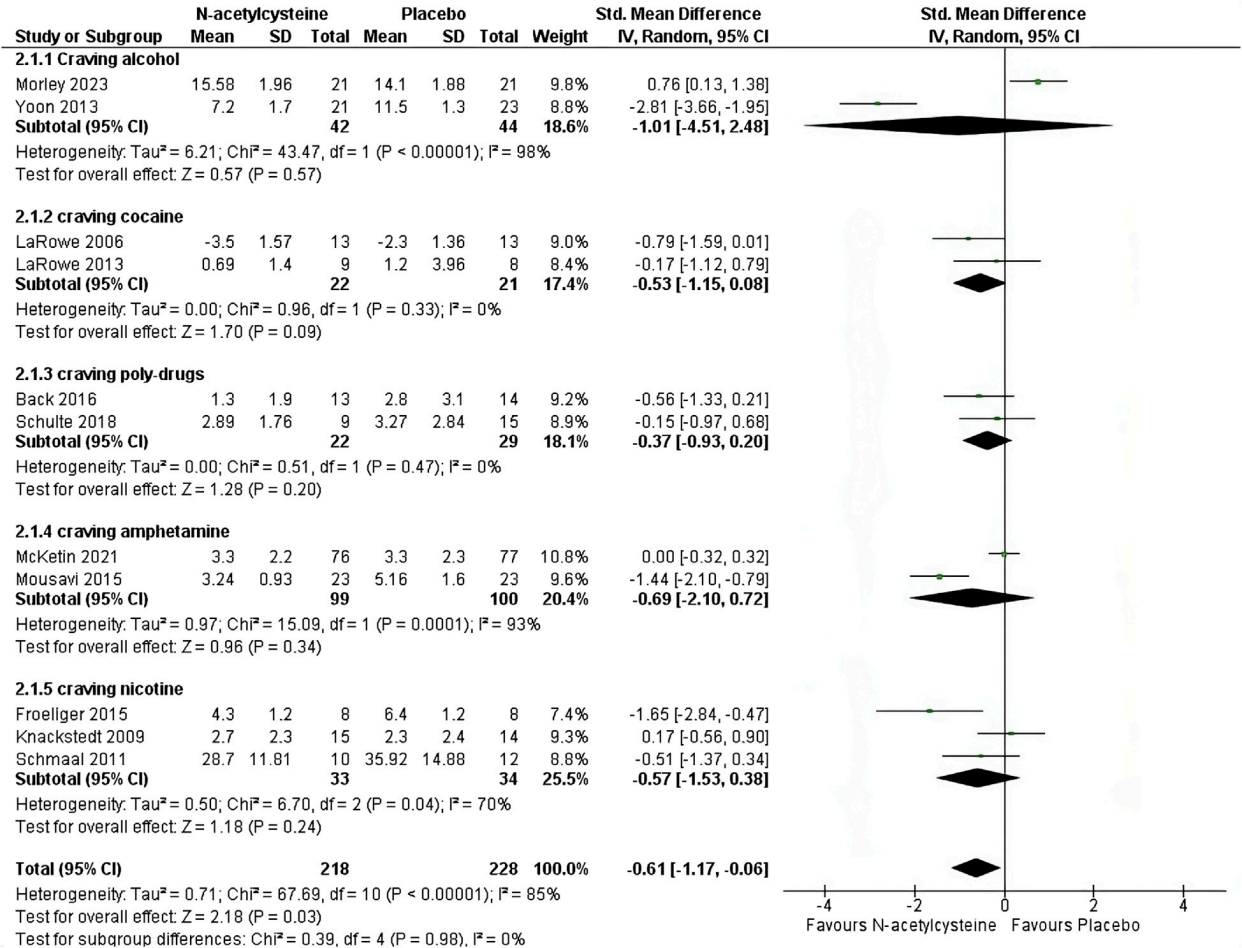
Forest plot reporting the differences in craving ratings in patients with SUD and treated with NAC as compared to Placebo. Abbreviation. SUD, Substances Use Disorder; NAC, N-acetylcysteine; CI, confidence interval; IV, Inverse Variance.

*Craving rating*: the analysis of data on craving represents our primary outcome. These data were assessed across 11 trials (LaRowe et al., 2013; Knackstedt et al., 2009; Morley et al., 2023; McKetin et al., 2021; Schulte et al., 2018; Back et al., 2016; Froeliger et al., 2015; Mousavi et al., 2015; Yoon, 2013; Schmaal et al., 2011; LaRowe et al., 2006) comprising a total of 446 patients. When multiple scales were used across trials to collect craving data, we considered the most commonly utilized scale among the analyzed studies. This approach ensured greater consistency in the data. Treatment with N-acetyl cysteine did lower the craving rating (SMD -0.61 (−1.17, −0.06), *p* = 0.03, I^2^ = 85%, Tau^2^ = 0.71) (Figure 2). The subgroup analysis according to the drug addiction (alcohol, cocaine, poly-drugs, amphetamine, nicotine) did not show differences (*p* = 0.98).

### 3.2 Secondary outcomes

#### 3.2.1 Withdrawal symptoms

The data on withdrawal symptoms are found in 5 trials (Knackstedt et al., 2009; McKetin et al., 2021; Froeliger et al., 2015; Schmaal et al., 2011; LaRowe et al., 2006). Among these, 3 trials (Knackstedt et al., 2009; Froeliger et al., 2015; Schmaal et al., 2011) assessed the effect of NAC on nicotine dependence. Different scales were used to assess withdrawal symptoms across these trials: specifically, Schmall, 2011 (Schmaal et al., 2011) and Knackstedt, 2009 (Knackstedt et al., 2009) used the MNWS (Minnesota Nicotine Withdrawal Symptoms), while Froeliger, 2015 (Froeliger et al., 2015) used a modified version of the SJWQ (Schiffman-Jarvik Questionnaire). McKetin, 2021 (McKetin et al., 2021) evaluated withdrawal symptoms using the AWQ (Amphetamine Withdrawal Questionnaire), and LaRowe, 2006 (LaRowe et al., 2006) used the CSSA (Cocaine Selective Severity Assessment). The data were analyzed for 245 patients and showed no significant difference between treatment groups (SMD -0.18 (−0.43, 0.08), *p* = 0.17), with no heterogeneity detected among the studies (*p* = 0.56, I^2^ = 0%, Tau^2^ = 0.00).

#### 3.2.2 Adverse Events

of the 11 trials analyzed, 9 reported data on adverse effects. Among these, 4 trials (Morley et al., 2023; McKetin et al., 2021; Froeliger et al., 2015; LaRowe et al., 2006) provided data only on the number of adverse events between the two treatment groups. The trials that instead reported the number of subjects experiencing adverse events were 3 (Back et al., 2016; Mousavi et al., 2015; Schmaal et al., 2011). Two trials reported data on adverse events both in terms of the number of subjects involved and the total number of events (LaRowe et al., 2013; Yoon, 2013). Therefore, we decided to analyze data only from trials that reported data on subjects experiencing adverse events during treatments (LaRowe et al., 2013; Back et al., 2016; Mousavi et al., 2015; Yoon, 2013; Schmaal et al., 2011). No substantial difference was observed between the two treatment groups (RR = 1.06 (0.89–1.27), *p* = 0.52, I^2^ = 0%, Tau^2^ = 0.00). The forest plot of the outcome and the table of total adverse events can be found in the Supplementary Material. The most common adverse events were, in order of frequency, gastrointestinal symptoms (nausea, constipation, mild stomachache, heartburn), headache and dry mouth.

#### 3.2.3 Drop-out

Eight RCTs (LaRowe et al., 2013; Knackstedt et al., 2009; Morley et al., 2023; McKetin et al., 2021; Schulte et al., 2018; Back et al., 2016; Mousavi et al., 2015; Yoon, 2013) reported drop out, with data on 490 patients. The results are slightly in favor of the placebo but are not statistically significant (RR 1.17 (0.85, 1.61), *p* = 0.34; I^2^ = 0%, Tau^2^ = 0.00). The reasons for patient drop-out were not specified in all trials. However, where specified, patients did not drop-out due to reasons related to group membership. No one dropped out due to adverse events associated with NAC administration.

### 3.3 Risk of bias assessments and publication bias

The results of the assessment of risk of bias according to the RoB2 tool are reported as Supplementary Material. In particular, in terms of the overall evaluation in the risk of bias, seven RCTs were deemed at high risk and four had some concerns. Visual inspection of the funnel plots concerning the outcome (Supplementary Material) suggests potential publication bias.

## 4 GRADE of evidence and TSA

**FIGURE 3:**
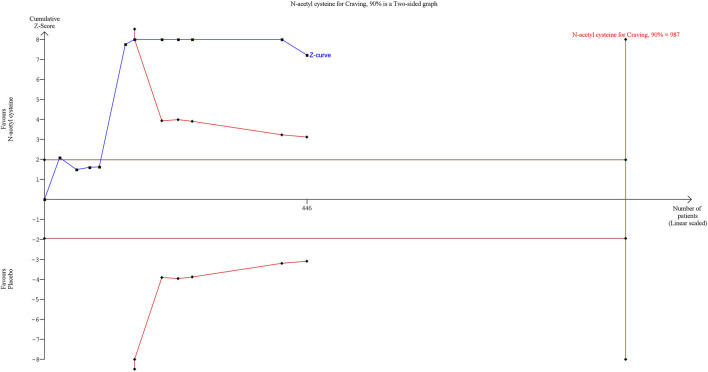
Trial Sequential Analysis on Craving ratings. Abbreviation. RE, random effect; RRR, relative risk reduction.

The results of assessment of the GRADE of evidence for the primary and secondary outcomes are reported in Supplementary Material. Due to the serious rating mainly in terms of risk of bias and indirectness of findings, the outcomes investigated were judged to have a low level of certainty. Trial Sequential Analysis (TSA) was conducted on the primary outcome using TSA software (Copenhagen Trial Unit, Centre for Clinical Intervention Research, Copenhagen), considering a type I error of 5% and a power of 90%. The Z-curve crossed the conventional boundary, indicating that a significant effect has been reached. However, the TSA revealed that the results are not yet robust, as the Z-curve did not cross the Required Information Size (RIS) boundary, with a ratio of patients recruited/needed of n = 446/987 (Figure 3). This suggests that the meta-analysis results may not be considered reliable due to an insufficient sample size. Therefore, further research is warranted.

## 5 Discussion

Substance use disorders (SUDs), including those involving cocaine, alcohol, nicotine, cannabis, amphetamines, and heroin, often require a multifaceted treatment approach. This typically combines pharmacological interventions, behavioral therapies, and social support mechanisms (Schwartz et al., 2022; Volkow and Blanco, 2023; Burnette et al., 2022; NIDA, 2019; NIDA, 2021a; NIDA, 2021b; NIDA, 2023; NIDA, 2024; Rigotti et al., 2022; Ghafouri et al., 2024; Moszczynska, 2021; ASAM national practice, 2020). Pharmacotherapy aims to alleviate withdrawal symptoms and cravings, while behavioral interventions, such as Cognitive Behavioral Therapy (CBT) and group therapy, focus on modifying substance-related behaviors. Social support, including family involvement and participation in support groups, plays a crucial role in the recovery process (Lohoff, 2022). Despite the availability of various treatment modalities, no single intervention is universally effective for all substance dependencies, highlighting the need for ongoing research to develop novel therapies with improved efficacy and tolerability profiles. In this context, N-acetylcysteine (NAC) emerges as a potentially valuable adjunctive treatment due to its cost-effectiveness and favorable tolerability profile, pending further validation of its efficacy (dos Santos Tenório et al., 2021).

Our meta-analysis of randomized controlled trials (RCTs) primarily investigated NAC’s efficacy in reducing craving ratings among individuals with SUD. Our findings support and extend the results of previous studies, including those by Duailibi et al. (2017) (Duailibi et al., 2017) and Chang et al. (2021) (Chang et al., 2021), which reported similar effects of NAC on addiction-related outcomes. However, our analysis is more current, incorporating newer data and a larger number of studies. This provides a more comprehensive evaluation of NAC’s efficacy and reflects the latest developments in the field. Therefore, our findings represent a significant advancement in understanding NAC’s role in addiction treatment.

Our analysis included studies involving polydrug users, such as those by Back et al. (2016) (Back et al., 2016) and Schulte et al. (2018) (Schulte et al., 2018). Despite the inclusion of these populations, our overall findings remained consistent. Initially, the effect size for craving reduction was SMD -0.61 (−1.17, −0.06) with a *p*-value of 0.03. Excluding studies involving polydrug users adjusted the effect size to −0.68 (−1.35, −0.01) with a *p*-value of 0.05. This adjustment indicates that while the effect size and significance were slightly influenced by the presence of polydrug users, the overall conclusion about NAC’s effectiveness in reducing craving remains valid. Thus, our meta-analysis supports NAC as an effective treatment for various substance use disorders.

Our findings show that NAC led to a reduction in craving ratings compared to placebo. However, it did not demonstrate significant differences in secondary outcomes, such as withdrawal symptoms, adverse events, or dropout rates. Several factors contribute to the limitations of our results.

First, our Trial Sequential Analysis (TSA) revealed that the information size required for robust conclusions was far from being met, with 446 patients enrolled across the RCTs, representing approximately 45% of the needed sample size (n = 987). Consequently, the current findings regarding NAC’s efficacy in reducing craving in SUD cannot be considered definitive. Second, the Grading of Recommendations Assessment, Development, and Evaluation (GRADE) of evidence indicated low certainty due to the risk of bias in several studies and the indirectness of the findings.

Another potential limitation of this meta-analysis is the lack of a sex-disaggregated analysis. While the studies included in our review did not consistently provide results broken down by sex, this gap underscores the need for future research to more thoroughly consider Sex as a Biological Variable (SABV).

An additional limitation of our study was the inability to analyze certain outcomes initially outlined in our protocol due to insufficient data in the included RCTs. Specifically, the parameter of substance-free days, which reflects the length of abstinence, could not be assessed. This metric is closely related to withdrawal symptoms and would have provided a deeper understanding of the efficacy of NAC.

Our analysis demonstrated a statistically significant reduction in craving ratings with NAC treatment. However, the incorporation of recent studies, particularly those by Morley (2023) and McKetin (2021), has introduced additional variability in the outcomes compared to earlier investigations. This underscores the complexity of addiction treatment and suggests the need for ongoing research to better understand these variations.

Furthermore, our subgroup analyses exploring different drug types (alcohol, cocaine, poly-drugs, amphetamines, nicotine) did not show significant differences (*p* = 0.98), indicating that NAC’s effect on craving reduction may be consistent across various substances. This consistency in effect across diverse substance use contexts reinforces the need for further investigation to clarify how NAC may impact different aspects of addiction and to optimize its therapeutic use.

An important consideration is the variation in craving rating assessment scales used across studies (e.g., PACS, Penn Alcohol Craving Scale (Flannery et al., 1999); CEQ, Craving Experience Questionnaire (May et al., 2014); VAS, Visual Analogue Scale (Wewers and Lowe, 1990); CCQ-Brief, Cocaine Craving Questionnaire-Brief (Sussner et al., 2006); BSCS, Brief Substance Craving Scale (Mezinskis et al., 2001); QSU-B, Questionnaire for Smoking Urges-Brief (Cox et al., 2001)).

Furthermore, the duration of intervention varied between studies, ranging from 0.4 to 12 weeks. Although the dosage of NAC was largely consistent across most RCTs (2,400 mg/day) (Baker et al., 2003); (Page et al., 2021; Sanfilippo et al., 2021; Kang, 2021; LaRowe et al., 2013; Knackstedt et al., 2009), a few studies employed different dosages (600 mg/day, 3,600 mg/day, and 1,200 mg/day) (Mousavi et al., 2015; Yoon, 2013; Schmaal et al., 2011; LaRowe et al., 2006). For example, LaRowe (2013) (LaRowe et al., 2013) compared two different NAC dosages (1,200 mg/day and 2,400 mg/day) with a placebo.

The rationale for using NAC in SUD patients stems from its potential to rebalance glutamate levels in the central nervous system (CNS) and modulate neurotransmitters such as glutamate and dopamine (Baker et al., 2003). Changes in glutamate homeostasis are observed in SUD patients, with reduced basal concentrations and increased levels during craving (Engeli et al., 2021; Smaga et al., 2021).

Despite these promising aspects, several limitations in interpreting our findings must be acknowledged, including heterogeneity among studies regarding design and participant characteristics, and reliance on self-reported measures which may introduce bias. Future research should address these limitations by employing standardized protocols and outcome measures. Longitudinal studies assessing the sustained effects of NAC treatment and investigations into its mechanisms of action could provide valuable insights into its therapeutic potential. Moreover, comparative effectiveness trials comparing NAC with existing pharmacotherapies may elucidate its role within the broader landscape of addiction treatment modalities.

## 6 Conclusion

In conclusion, our study underscores the promising role of N-acetyl cysteine as a potential adjunctive treatment for drug addiction, particularly in reducing craving, while further research is warranted to elucidate its effects on withdrawal symptoms and long-term outcomes, contributing to the growing body of evidence supporting the therapeutic utility of NAC in addiction management.

## Data Availability

The original contributions presented in the study are included in the article/Supplementary Material, further inquiries can be directed to the corresponding author.
