# Supplementary material for: Effect of N-acetylcysteine on craving in substance use disorders (SUD): a meta-analysis of randomized controlled trials
Source: Front Pharmacol. 2024 Sep 6;15:1462612. doi: 10.3389/fphar.2024.1462612 (PMC11412889; doi:10.3389/fphar.2024.1462612)
Supplement: Supplementary file 1 [file DataSheet1.docx]

Supplementary Material

1. **Supplementary Figures and Tables**
   1. **Supplementary Figures**


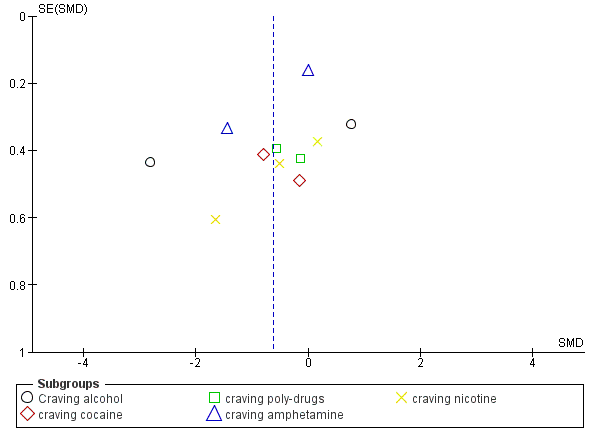


Supplementary Figure 1 - Funnel plot of primary outcome – Craving rating – in primary analysis to evaluate the existence of publication bias.


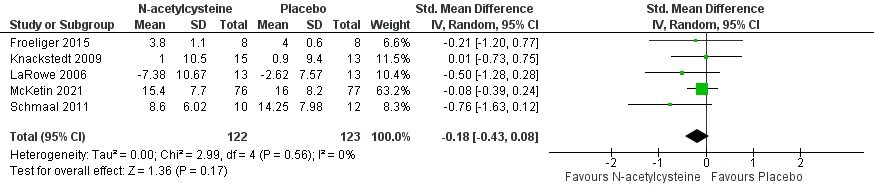


Supplementary Figure 2 - Forest plot of secondary outcome reporting the Withdrawal symptoms in patients admitted treated with NAC as compared to Placebo. *Abbreviation*: NAC, N-acetylcysteine; CI, confidence interval; IV: Inverse Variance.


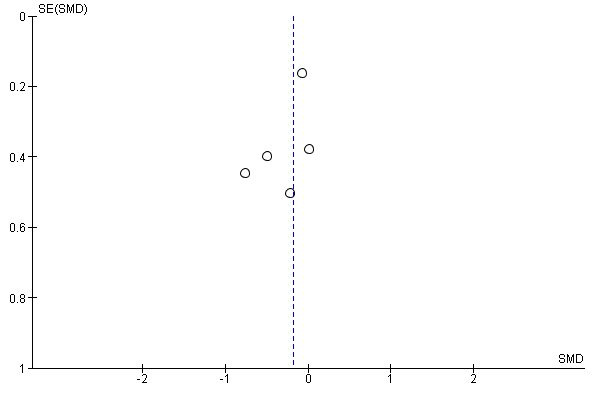


Supplementary Figure 3 - Funnel plot of secondary outcome – WS – in primary analysis to evaluate the existence of publication bias. *Abbreviation*: WS, Withdrawal Symptoms.


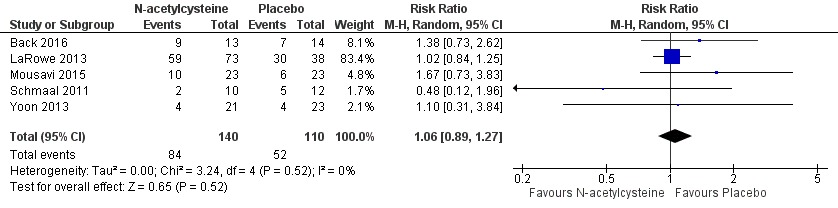


Supplementary Figure 4 - Forest plot of secondary outcome reporting the Adverse events in patients treated with NAC as compared to Placebo. *Abbreviation*: CI, Confidence Interval; M-H: Mantel-Haenszel; NAC, N-acetylcysteine.


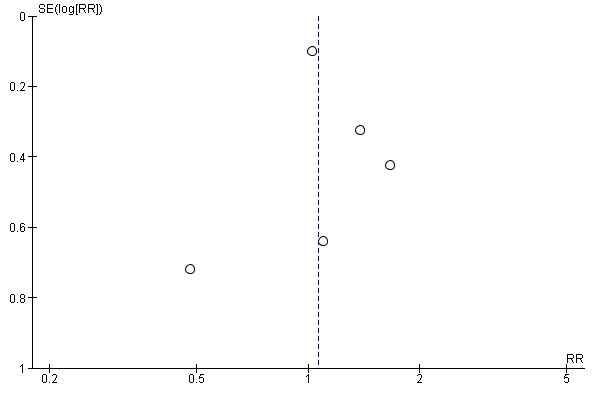


Supplementary Figure 5 - Funnel plot of secondary outcome – Adverse events – in primary analysis to evaluate the existence of publication bias.


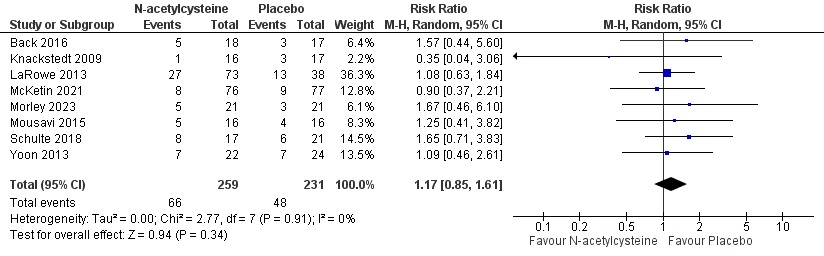


Supplementary Figure 6 - Forest plot of secondary outcome reporting the Drop Out in patients treated with NAC as compared to Placebo. *Abbreviation*: CI, Confidence Interval; M-H: Mantel-Haenszel; NAC, N-acetylcysteine.


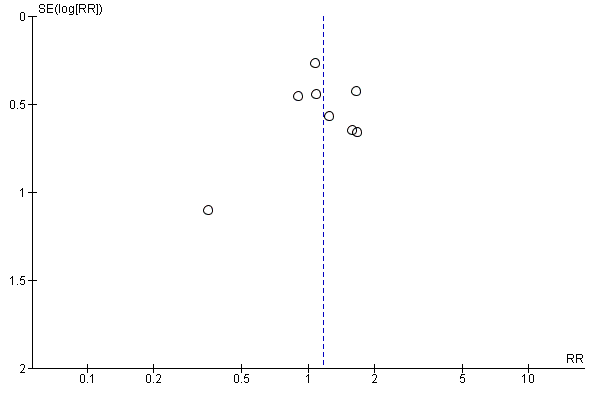


Supplementary Figure 7 - Funnel plot of secondary outcome – Drop Out – in primary analysis to evaluate the existence of publication bias.


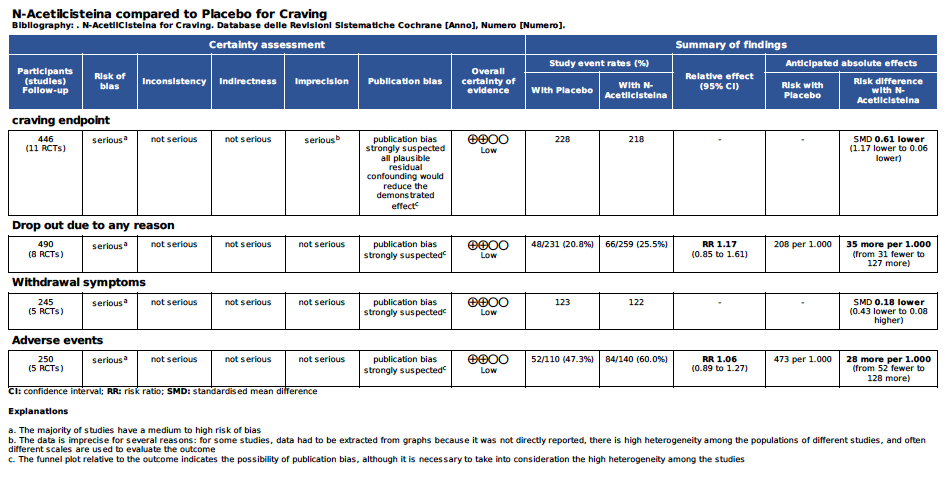
**Supplementary Figure 8** – GRADE

- 1. **Supplementary Tables**

| *Population* | Patients with substances use disorders (SUD) |
| --- | --- |
| *Intervention* | N-acetylcysteine, any dosage |
| *Comparison* | Placebo |
| *Outcomes* | Primary outcome: craving rating  Secondary outcome: withdrawal symptoms, adverse events (number of non-serious adverse events, number of serious adverse events) and dropouts. |
| *Study design* | RCT |

Supplementary Table 1 - PICOS approach for selecting clinical studies in the systematic search and meta-analysis. *Abbreviation*: PICOS, population, intervention, comparison, outcome, study design.

| **Adverse Events (n)** | | | |
| --- | --- | --- | --- |
|  | **NAC** | **Placebo** | **Total** |
| *Morley, 2023* | 7 | 6 | 13 |
| *LaRowe, 2006* | 20 | 13 | 33 |
| *LaRowe, 2013* | 85 | 54 | 139 |
| *Yoon, 2013* | 7 | 5 | 12 |
| *Back, 2016* | / | / | 31 |
| *McKetin, 2021* | 102 | 108 | 210 |
| *Mousavi, 2015* | 10 | 6 | 16 |
| *Froeliger, 2015* | 5 | 12 | 17 |
| *Schmall, 2011* | 5 | 2 | 7 |

**Supplementary Table 2 –** Number of adverse events in patients treated with NAC as compared to Placebo and total*. Abbreviation:* NAC*, N-acetylcysteine.*

Supplementary Table 3 - Risk of bias of RCTs evaluated in primary analysis. *Abbreviation*: RCT, Randomized Clinical Trial.
